# Characterisation of *Campylobacter* spp. Isolated from Poultry in KwaZulu-Natal, South Africa

**DOI:** 10.3390/antibiotics9020042

**Published:** 2020-01-21

**Authors:** Stephanie Pillay, Daniel G. Amoako, Akebe L. K. Abia, Anou M. Somboro, Christiana O. Shobo, Keith Perrett, Linda A. Bester, Sabiha Y. Essack

**Affiliations:** 1 Antimicrobial Research Unit, College of Health Sciences, University of KwaZulu-Natal, Durban 4000, South Africa; stephaniepillay14@gmail.com (S.P.); lutherkinga@yahoo.fr (A.L.K.A.); anou.somboro@gmail.com (A.M.S.); Wunmex6@yahoo.co.uk (C.O.S.); essacks@ukzn.ac.za (S.Y.E.); 2 Biomedical Resource Unit, College of Health Sciences, University of KwaZulu-Natal, Durban 4000, South Africa; besterl@ukzn.ac.za; 3 Epidemiology Section, KwaZulu-Natal, Agriculture & Rural Development—Veterinary Service, Pietermaritzburg 3200, South Africa; Keith.Perrett@kzndard.gov.za

**Keywords:** farm-to-fork, antibiotic resistance, virulence, campylobacter, clonality, poultry, South Africa

## Abstract

This study investigated the antibiotic resistance, virulence profiles, and clonality of *Campylobacter jejuni* and *Campylobacter coli* isolated from an intensive poultry farming system in KwaZulu-Natal, South Africa. Following ethical approval, samples were collected over six weeks using the farm-to-fork approach. *Campylobacter* spp. were identified using culture, confirmed and differentiated to species level by PCR, and subjected to antibiotic susceptibility testing. Selected antibiotic resistance (and mutations) and virulence genes were screened by PCR and confirmed by DNA sequencing. Genetic relatedness amongst the isolates was ascertained using pulsed-field gel electrophoresis. In all, 105 isolates were confirmed as belonging to both *Campylobacter* *coli* (60; 57%) and *C. jejuni* (45; 43%). The highest resistance was recorded against erythromycin and clindamycin. The *gyrA* mutation, A20175C/A2074G point mutation, *tet(O)*, and *cmeB*, all associated with antibiotic resistance, were detected. All the virulence genes (*pldA*, *ciaB*, *cdtA, cdtB*, *cdtC*, *dnaJ*, except for *cadF*) were also detected. Isolates were grouped into five pulsotypes displaying 85% similarity, irrespective of their resistance profiles. The numerous permutations of clonality, antibiotic resistance, and virulence profiles evident in *Campylobacter* spp. pose a challenge to food safety and necessitate a comprehensive understanding of the molecular epidemiology of this organism to decrease its spread in the food chain.

## 1. Introduction

Poultry is one of the world’s largest sources of meat, and production usually takes less than six weeks [1]. The use of antibiotics as growth promoters improves gut health but simultaneously exerts selection pressure for the development of resistance [2]. For example, resistant *Campylobacter* spp. are on the rise due to the continuous use of antibiotics in poultry. Human campylobacteriosis is classified as mild enteritis and is usually self-limiting; however, in extreme cases, antibiotic treatment is necessary [3].

*Campylobacter* spp. are leading foodborne pathogens, responsible for approximately 550 million cases of diarrhoea globally with *Campylobacter coli* and *Campylobacter jejuni* being the most frequently implicated aetiological sub-species [4]. The epidemiology of *Campylobacter* spp. is multifaceted due to the wide distribution of the bacteria, their genetic variability, and interactions with their host [5]. The primary sources of infection include the ingestion of either raw or uncooked meat products, in addition to contaminated drinking water and, unpasteurized milk [6]. Avian species, more specifically chickens, constitute a significant reservoir for the transmission of *Campylobacter* spp. due to their high body temperature, which is the optimum growth condition for this pathogenic bacterium [7]. *Campylobacter* spp. can colonise the caeca of chickens in extremely high numbers, although the chickens rarely show signs of disease as compared to campylobacteriosis in humans, which can be fatal [8]. Chickens are exposed to *Campylobacter* during the breeding process on farms by ingestion of contaminated water or by contact with faeces from other contaminated birds, and this is termed horizontal transmission.

Although several virulence genes may contribute to some extent to colonisation ability, others may contribute to the development of an asymptomatic carrier state. In the case of infection, these include virulence factors that contribute to the pathogenicity of the microorganism, as well as persistent infection as a result of antibiotic-resistant strains [5]. Antibiotics that are usually prescribed for the treatment of campylobacteriosis include tetracycline, macrolides (erythromycin), and fluoroquinolones (ciprofloxacin), with aminoglycosides (gentamicin) prescribed for systemic infections [5]. Most of these antibiotic classes have analogues used in veterinary practices for growth promotion, prophylaxis, metaphylaxis and treatment in food animals, for example, tylosin and kitasamycin (macrolides), enrofloxacin (quinolones) and doxycycline (tetracyclines) [9]. Resistance to antibiotics such as fluoroquinolones, tetracyclines, β-lactams, aminoglycosides, and macrolides has been reported in *Campylobacter* spp. [10]. Moreover, virulence factors play a significant role in establishing infection. Common virulence factors in *Campylobacter* spp. include those responsible for adhesion, invasion, toxin production, and thermo-tolerance [11].

South Africa is Africa’s largest producer of chicken, with an estimated 2.152 million tonnes of poultry meat consumed in the country per year [12,13]. However, there is currently a paucity of data on the molecular epidemiology of *Campylobacter* in poultry in South Africa. Furthermore, no studies have been conducted to investigate this along the “farm-to-fork” continuum advocated by the World Health Organization Advisory Group on Integrated Surveillance of Antimicrobial Resistance (WHO-AGISAR) as one of the appropriate methods in monitoring foodborne pathogens [14]. There is, therefore, a need to ascertain the antibiotic resistance of *Campylobacter* spp. in poultry to encourage implementation of measures for its containment. This study describes the antibiotic resistance, virulence profiles and genetic relationships of *C. jejuni* and *C. coli* isolated from farm-to-fork in an intensive poultry farming system in uMgungundlovu District, KwaZulu-Natal, South Africa.

## 2. Results

### 2.1. Prevalence of Campylobacter *spp.*

Putative *Campylobacter* spp. isolates were recovered from 191 positive samples, which included water samples collected at farm level, the abattoir (carcass rinsate) and retail meat products (neck and thigh swabs and whole carcass rinsate). Samples analysed from weeks one to five did not contain Campylobacter spp.; this included faeces, litter, truck, and crate samples. Of the 191 putative *Campylobacter* isolates, 176 were confirmed as belonging to the genus *Campylobacter* using PCR, of which 105 were confirmed as *Campylobacter* spp., with 60 (57%) further identified as *C. coli* and the remaining 45 (43%) as *C. jejuni*. The distribution of the isolates relative to the sampling site included: farm (week 5)—14, abattoir—72, and retail meat products—19.

### 2.2. Antibiotic Susceptibility

**Figure 1:**
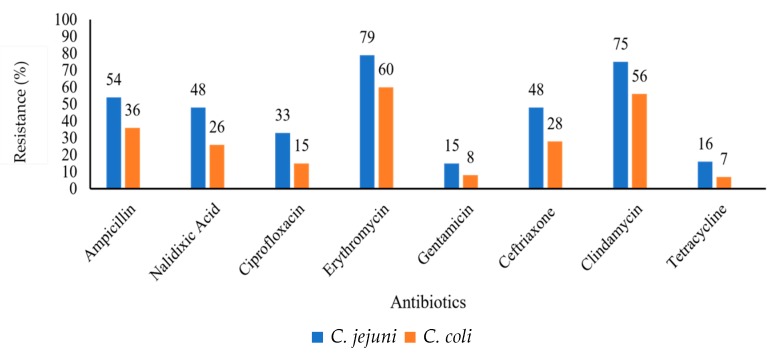
Graphical representation of resistance (%) of total *C. jejuni* (*n* = 45) and *C. coli* (*n* = 60) isolates against a panel of antibiotics.

The antibiotic susceptibility profiles of *C. jejuni* and *C. coli* showed a high percentage resistance to erythromycin (79% and 60%, respectively) and clindamycin (75% and 56%, respectively). The lowest percentage resistance shown for both isolates were to gentamicin (15% and 8%) and tetracycline (16% and 7%) for *C. jejuni* and *C. coli*, respectively (Figure 1).

**Figure 2:**
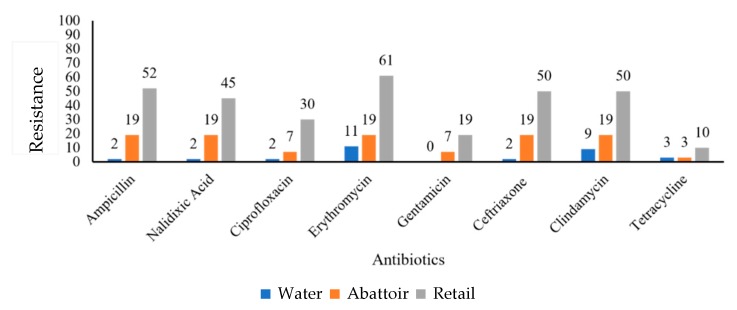
Graphical representation of resistance (%) of *C. jejuni* (*n* = 45) isolates obtained from various sources against a panel of antibiotics.

Regarding *C. jejuni*, retail meat product isolates showed the highest percentage resistance to erythromycin (61%), ceftriaxone (50%) and clindamycin (50%) as compared to the isolates obtained from the abattoir. All the isolates recorded a 19% resistance to ampicillin, nalidixic acid, erythromycin, ceftriaxone, and clindamycin. Similarly, 11% of water isolates showed resistance to erythromycin (Figure 2).

**Figure 3:**
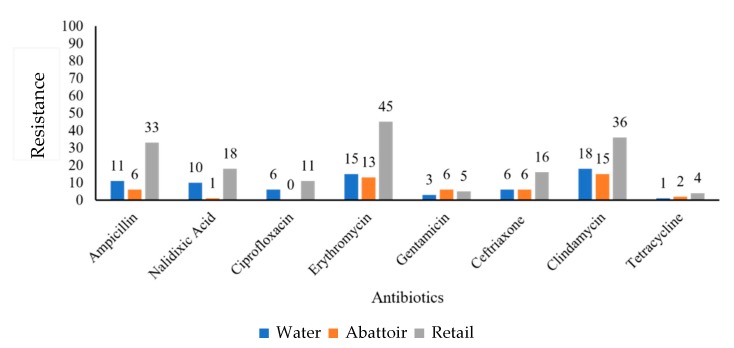
Graphical representation of resistance (%) of *C. coli* (*n* = 60) isolates obtained from various sources against a panel of antibiotics.

*C. coli* isolates obtained from retail meat products showed high resistance to erythromycin (45.1%) and clindamycin (36%), whereas abattoir and water samples showed high resistance to clindamycin of 15% and 18%, respectively (Figure 3).

*C. jejuni* displayed 15 different antibiotic resistance phenotypic profiles as compared to *C. coli,* which exhibited 21 profiles. The most common profiles in both sub-species were the resistance to six antibiotics (AMP-NAL-CIP-ERY-CRO-CLI) and the resistance to two antibiotics (ERY-CLI) as seen in Table 1.

The detail antibiogram of each isolate, including the isolates name and the point of isolation, are presented in Table S1 (Supplementary Materials).

### 2.3. Antibiotic Resistance Genes

Antibiotic resistance genes were screened in isolates that presented phenotypic resistance to the selected antibiotics. In all, 6/10 of *C. jejuni* and 5/10 *C. coli* showed a mutation at the Thr-86-Ile region in the quinolone-resistance-determining region (QRDR) of the *gyrA* of fluoroquinolone-resistant isolates. Also, the transitional mutations A2075G and A2074C in the *23S rRNA* genes were amplified in 7/10 *C. coli,* and 6/10 *C. jejuni* isolates. *Campylobacter* multidrug resistance gene (*cmeB*) was amplified in 30 (67%) *C. jejuni* and 45 (75%) *C. coli* isolates. All isolates resistant to tetracycline carried the *tet(O)* gene. These samples were sequenced, and all showed similarity with known *gyrA*, *tet(O),* and macrolide resistance genes of *C. jejuni* and *C. coli* sequences in GenBank.

### 2.4. Virulence Factors

**Figure 4:**
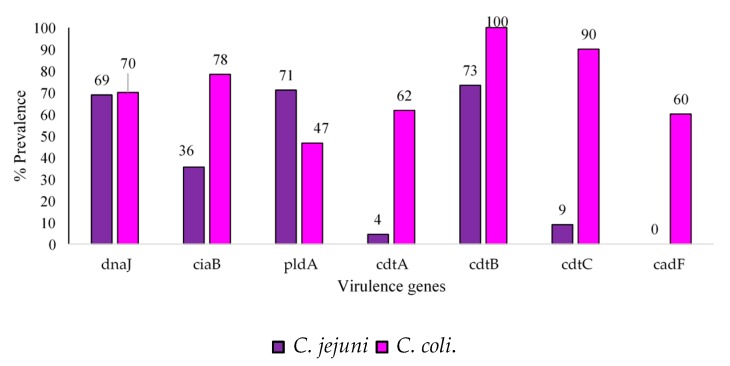
Prevalence (%) of virulence genes in total *C. jejuni* (*n* = 45) and *C. coli* (*n* = 60) isolates.

It was found that 100% and 90% of *C. jejuni* and *C. coli* isolates were positive for the *cdtB* and *ctdC* genes, respectively (Figure 4).

**Figure 5:**
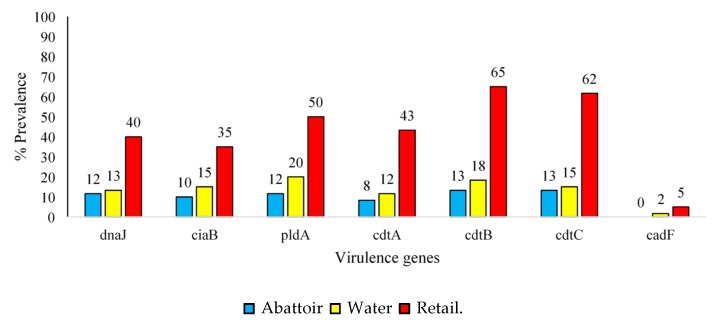
Prevalence (%) of virulence genes detected in *C. coli* (*n* = 60) isolated from abattoir, water, and retail meat product samples.

In addition, 62% of *C. coli* isolates tested positive for the *cadtA* gene, whereas only 4% of *C. jejuni* isolates were positive for the *cdtA* gene. Regarding *C. coli* samples, 65% of retail meat product isolates, 20% of water isolates and 13% of abattoir isolates were positive for the *cdtB*, *pldA* and *cdtC* genes, respectively (Figure 5).

**Figure 6:**
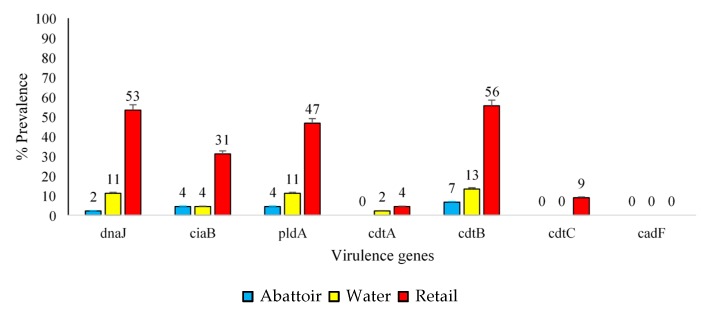
Prevalence (%) of virulence genes detected in *C. jejuni* (*n* = 45) isolated from abattoir, water, and retail meat product samples.

Moreover, only 35% of retail meat product isolates, 2% of water isolates and 0% of abattoir isolates were positive for *ciaB* and the *cadF* gene. The *CdtB* gene was found to be the most common in *C. jejuni* isolates obtained from retail meat product (35%), abattoir (13%) and water (7%) samples (Figure 6). The *cadF* gene was not present in any of these isolates.

### 2.5. Genetic Relationship by Pulsed-Field Gel Electrophoresis

**Figure 7:**
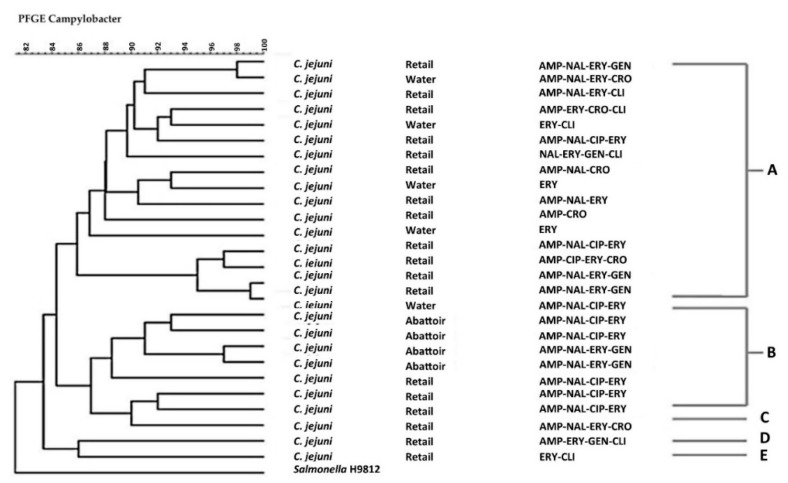
Pulsed-Field Gel Electrophoresis (PFGE) *Sma*1 genotypes generated from *C. jejuni* isolates obtained from different sample sources that displayed different antibiotic-resistant profiles. AMP = Ampicillin, NAL = Nalidixic acid, CIP = Ciprofloxacin, ERY = Erythromycin, TET = Tetracycline, CRO = Ceftriaxone, GENT = Gentamicin, CLI = Clindamycin. The letters A–E indicate the clusters of isolates with *Salmonella* Braenderup H9812 used as a control.

**Figure 8:**
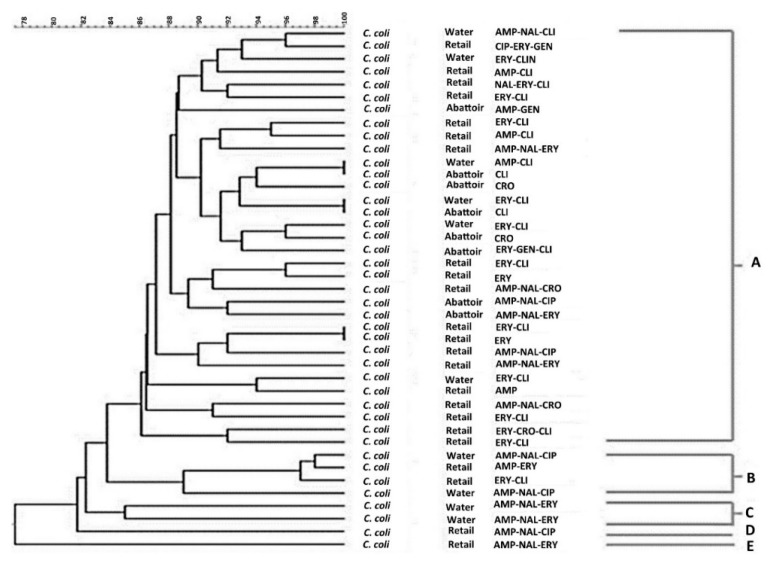
PFGE *Sma*1 genotypes generated from *C. coli* isolates obtained from different sample sources that displayed different antibiotic-resistant profiles. AMP = Ampicillin, NAL = Nalidixic acid, CIP = Ciprofloxacin, ERY = Erythromycin, TET = Tetracycline, CRO = Ceftriaxone, GENT = Gentamicin, CLI = Clindamycin. The letters A–E indicate the clusters of isolates with *Salmonella* Braenderup H9812 used as a control.

The Pulsed-Field Gel Electrophoresis (PFGE) profiles and the dendrogram of *C. jejuni and C. coli* isolates are shown in Figure 7 and Figure 8, respectively.

Forty-one *C. coli* isolates and 27 *C. jejuni* isolates were grouped into five pulsotypes designated A–E displaying 85% similarity irrespective of their resistant profiles. Of note, *C. coli* and *C. jejuni* showed a similar clonal trend.

## 3. Discussion

### 3.1. Prevalence of Campylobacter *spp.*

This study involved the characterisation of *Campylobacter* spp. from farm-to-fork in an intensive poultry farming system located in the uMgungundlovu District of KwaZulu-Natal, South Africa. This study was conducted using the farm-to-fork approach as recommended by the World Health Organization Advisory Group on the Integrated Surveillance of Antimicrobial Resistance (WHO-AGISAR) in collaboration with the Food and Agriculture Organization (FAO), and World Organization for Animal Health (OIE) [14]. The approach involves sampling from the farm, through the transport system, to the processing units (abattoir), and finally to the fork (the retail point). From an epidemiological perspective, this would aid in determining the sources and causes of an illness (and antibiotic resistance), especially during an outbreak. This approach has been implemented to show the potential movement of resistant bacteria across the poultry food production chain in South Africa [15,16].

No presumptive *Campylobacter* spp. isolates were observed during weeks one to five as similarly described in an observational study by Newell and Fearnley [17], who demonstrated that *Campylobacter* spp. colonisation in broiler chickens occurs after approximately four weeks because the intestinal niche of the chicks undergoes physiological changes during the first three to four weeks of life. Even though chicks are exposed to *Campylobacter* spp. in the environment, the maturing mucosal immunity results in shifts in microflora during the first 3–4 weeks, which is known as “lag phase” [7,17].

*C. coli* (56%) was more prevalent than *C. jejuni* (42%) as this sampling was conducted during winter months. Willis and Murray [18] stated that there were higher recovery rates of *C. jejuni* in warmer months of the year. Similarly, Maćkiw et al. [10], conducted a study between January 2008 and December 2009 where chicken meat and giblets sampled from retail trade in Poland demonstrated the presence of *C. coli* in 108 samples out of 143, whereas *C. jejuni* was found in 35 of the samples. A baseline survey conducted in the United Kingdom in 2008 reported the prevalence of *Campylobacter* spp. in broiler flocks and carcasses from four different poultry farms indicating a significant association with highly contaminated carcasses in all four farms and farm locations with a higher prevalence of *C. jejuni* as compared to *C. coli* due to slaughter taking place in the summer months [19].

### 3.2. Virulence Factors

Figure 5 and Figure 6 demonstrate that many isolates originating from these sources, especially retail meat products, had pathogenic potentials for humans [20,21]. Contamination of retail meat products occurs from the intestinal contents during the pre- and post-processing phase leading to outbreaks in humans. The pathogenicity of *Campylobacter* spp. is mediated by several virulence factors. *cdtA, cdtB,* and *cdtC* encode for a protein that releases cytotoxins that damage the nuclear DNA. Cell cycle arrest and cell death could also be caused by the translocation of the *cdtB* gene to the nucleus of the cells inducing genotoxic effects on the host DNA [22]. A high prevalence of *cdtA* (61%) and *cdtC* (90%) was observed in *C. coli* isolates only in retail meat products. As stated by Lapierre et al., [23], the presence of a single *cdt* gene will not influence the virulence and pathogenicity of *Campylobacter*. Instead, all three *cdt* genes need to be present in a cluster, thus releasing a functional cytotoxin enhancing the bacterial virulence mechanisms. In addition to cytotoxic effects, invasion and adherence are also facilitated by *cdt* which is imperative for the release of interleukin-8 (IL-8) by the intestinal cells in vitro, thus contributing to the host mucosal inflammatory response produced in response to *Campylobacter* spp. infection [8]. Wieczorek [24] previously reported a higher prevalence of *cdtA*, *cdtB* and *cdtC* in *C. coli* (92.5%) compared to *C. jejuni* (14.6%) in meat products, similar to this study. Also, the *ciaB* gene (*Campylobacter* invasive antigen B), which aids in the translocation of *Campylobacter* [25], had a lower prevalence in abattoir and water samples compared to the retail meat products. Another important factor for colonisation of *Campylobacter* spp. within the intestines of chickens is the *pldA* gene, an outer membrane phospholipase A, encoding proteins that are associated with increased bacterial invasion of epithelial cells. The distribution of this gene was dissimilar among the two subspecies investigated as *C. jejuni* showed a higher prevalence (71%) compared to *C. coli* isolates (47%). The *dnaJ* gene enables *Campylobacter* spp. to cope under different physiological conditions [11]. *C. coli* and *C. jejuni* can respond to changes in temperature as they can colonise the avian gut [26]. Temperatures in the gut are at 42 °C whereas, in humans, it is 37 °C. However, during the transmission process, the temperatures of water, milk, or meat are at 4 °C [27]. The response to stress is carried out by heat shock proteins encoded by *dnaJ* [28]. The high prevalence of this gene found in retail products contributes to the pathogenicity of *Campylobacter* spp., as they can adapt and respond to different temperature changes given that most retail products are kept in freezers at supermarkets. If meat products are subsequently undercooked, this could lead to Campylobacteriosis in humans [29]. In a study conducted by Lapierre et al. [23], the presence of eleven virulence genes was tested in 528 *Campylobacter* spp. isolated from poultry retail meat products obtained from supermarkets in the metropolitan region of Chile. The *ciaB*, *pldA* and *dnaJ* genes were more prevalent in *C. jejuni* isolates compared to *C. coli* isolates. All three genes displayed a prevalence of over 60% in *C. jejuni* isolates, whereas the prevalence was less than 30% in *C. coli* isolates, similar to the findings of the present study.

As routine hygienic practice within the slaughterhouse, working surfaces are washed after the completion of a given flock and before the start of another. However, the personnel are not changed between flocks. Thus, there could be cross-contamination from one flock to another through the slaughterhouse personnel. This could have accounted for the higher prevalence of *Campylobacter* spp. and the associated virulence genes found in retail meat products compared to the other sampling sites. It is, therefore, imperative to conduct further studies involving the sampling of the slaughterhouse personnel to better understand the possibility of such cross-contamination.

### 3.3. Antibiotic Resistance Profiles and Resistance Determinants

A factor that must be considered for foodborne pathogenic species is their resistance to first-line antibiotics used in human therapy [23]. The increased resistance of bacteria to antibiotics has been associated with the continuous use of antibiotics either therapeutically, prophylactically, or as growth-promoting agents to maintain animal welfare in poultry production systems. This practice creates a potential risk for human health care based on the present knowledge of gene transfer and co-resistance [5]. First-line treatment options of human campylobacteriosis are macrolides (erythromycin), quinolones (ciprofloxacin) and tetracyclines. Veterinary analogues registered for use in poultry production are tylosin and kitasamycin (macrolides), enrofloxacin (quinolones) and doxycycline (tetracyclines) used in feed combinations or water supplements [30]. A study conducted in Western Cape, South Africa, 2010, reported a moderate percentage resistance (45.4%) to the tetracycline analogue, doxycycline, and high resistance to erythromycin (72.7%) for *C. coli* isolates. *C. jejuni* isolates showed a percentage resistance of 60% to doxycycline and 20% to erythromycin [29]. However, in this study, a higher percentage of resistance to erythromycin (79% and 60%) and a lower percentage resistance to tetracycline (16% and 7%) in both *C. jejuni* and *C. coli* isolates, respectively, was observed. The results of the previous study and those of this one call for the need for stringent antimicrobial surveillance programmes for the use of antibiotic analogues as growth promoters in food animal production.

Interestingly, isolates screened for mutations/genes causing resistance to ciprofloxacin, erythromycin, tetracycline, and the *cmeB* efflux gene shared similar results to a study conducted by Shobo et al. [31] on clinical samples from a private clinic in KwaZulu-Natal, South Africa. Also, a study conducted by Reddy and Zishiri [5] isolated *C. jejuni* and *C. coli* from the faeces of broiler chickens from a rural area in KwaZulu-Natal, South Africa, and reported detection rates of 50% for the *gyrA* mutation and 70% for the *tet(O)* gene.

Multidrug resistance to antibiotics was common amongst *C. jejuni* and *C. coli* isolates irrespective of the source (Table 1). In total, 15 and 22 different resistance profiles were observed for *C. jejuni* and *C. coli*, respectively, with most isolates showing resistance to six out of the eight antibiotics tested, that is, 11 *C. jejuni* isolates and eight *C. coli* isolates. Several *C*. *jejuni* and *C. coli* isolates displayed resistance to the macrolides, erythromycin, and clindamycin.

### 3.4. Genetic Relatedness of Isolates

Further analysis was done to observe the genetic diversity of these isolates based on their antibiotic resistance profiles. Pulsed-field gel electrophoresis (PFGE), which is the gold standard technique for strain typing, was used to predict the genetic relatedness of *C. jejuni* and *C. coli* isolates. The analysis revealed five pulsotypes A–E, which did not correlate with their resistance profiles for *C. jejuni* and *C. coli* isolates. The isolates were clustered at a similarity of 85% when analysed by *SmaI* PFGE. Figure 7 and Figure 8 show that 89% (24/27) and 90% (37/41) of *C. jejuni* and *C. coli* isolates were clustered into two major PFGE types, indicating a possibility of genetic relatedness amongst the *C. jejuni* and *C. coli* isolates, which were present between the abattoir, water and retail meat product samples irrespective of their resistance profiles. This was also evident in Figure 8, which shows that 90% (37/41) of *C. jejuni* isolates were clustered into two major clusters, A and B, indicating that similar *C. jejuni* strains were found in the water, abattoir and retail meat product samples irrespective of their resistance profiles. These results correlate with the results obtained by Denis et al. [32] who analysed the diversity of PFGE profiles of *C*. *jejuni* and *C. coli* from 26 free-range broiler chickens in France between 2003 and 2004. It was found that *C. jejuni* and *C. coli* isolates obtained from the different flocks were genetically related when grouped into clusters of 80% similarity. However, further studies that involve the use of more resolute typing approaches, such as whole genome-sequencing (WGS), will be needed to substantiate this claim.

## 4. Materials and Methods

### 4.1. Ethical Approval

Ethical approval was received from the Animal Research Ethics Committee (Reference: AREC 073/016PD) and the Biomedical Research Ethics Committee (Reference: BCA444/16) of the University of KwaZulu-Natal. The study was further placed on record with the South African National Department of Agriculture, Forestry, and Fisheries (Reference: 12/11/1/5 (879)). All information obtained from the farm was kept confidential as part of the memorandum of understanding (MOU) between the Antimicrobial Research Unit (ARU) and the farm.

### 4.2. Sampling Procedure

Samples were collected from an intensive poultry farming system, Farm A, in KwaZulu-Natal, South Africa, from 4 sites including the farm, transport (trucks), abattoir and from retail products along the farm-to-fork continuum as previously described [15]. A total of 384 samples were collected weekly over 6 weeks (August to September 2017) and consisted of litter, faeces and water samples (collected from the washing of the poultry house) at farm level. Truck and crate swabs were also collected during the transportation of the birds to the slaughterhouse. At the abattoir, carcass rinsate, caecal samples, and carcass swabs and retail meat products (neck, thigh, and whole carcass rinsate) were collected. Samples were directly inoculated into blood-free *Campylobacter* broth (Fluka Analytical, Kolkata, India), and transported to the laboratory for further processing.

### 4.3. Isolation and Identification of Campylobacter *spp.*

All samples were analysed within 6 h from collection. To obtain pure cultures, samples were processed as previously described by Shobo et al. [31]. Briefly, to confirm the purity of the culture, samples incubated in broth were filtered through a 0.47 µM mixed cellulose ester filter (Merck Millipore, Ireland) onto *Campylobacter* blood-free selective agar base (Oxoid, Hampshire, UK), supplemented with charcoal cefoperazone deoxycholate (CCD) agar-selective supplement (Oxoid, Hampshire, UK). Approximately 500 µL of the inoculum was evenly and aseptically distributed over the filter. Once the liquid had been filtered through, forceps were used to aseptically remove the filter. Plates were incubated at 37 °C in a microaerophilic atmosphere (CampyGen; Oxoid, UK) for 48 h. This was followed by sub-culturing on Tryptose Blood Agar Base (Biolab, Modderfontein, South Africa) supplemented with 5% defibrinated sheep blood and incubated at 37 °C for 42 h in a microaerophilic atmosphere. Susceptibility to nalidixic acid (30 µg Oxoid, UK) and cephalothin (30 µg Oxoid, UK) was also ascertained [33].

### 4.4. DNA Extraction

*Campylobacter* spp. isolates were cultured onto Mueller–Hinton agar (Oxoid, UK) supplemented with 5% defibrinated sheep blood and incubated as before. Template DNA was extracted for PCR using the boiling method as previously described [34]. Positive *Campylobacter* spp. controls were also prepared by isolating DNA from reference strains, *C. jejuni* ATCC 33560 and *C. coli* ATCC 33559, which were incubated under the same conditions and subjected to the same DNA extraction method. The Nanodrop 2000 UV-Vis Spectrophotometer (ThermoFisher Scientific, Lenexa KS, USA) was used to ascertain the concentration and quality of the isolated DNA [35].

### 4.5. Molecular Confirmation Of Isolates

*Campylobacter* genus and species were confirmed using real-time polymerase chain reaction (RT-PCR). Primers (Table 2) used for the amplification were purchased from Inqaba Biotechnical Industries (Pty) Ltd. Pretoria, South Africa.

The reaction was carried out in a total volume of 20 µL made up of 10 µL of a New England Biolabs^®^ 2× Luna^®^ Universal qPCR master mix (Inqaba Biotechnical Industries (Pty) Ltd., Pretoria, South Africa ), 0.5 µL of each primer (final concentration 0.5 µM), 5 µL of template DNA and 1 µL of nuclease-free water. The PCR conditions were as previously optimised by Chukwu et al. [38] with slight modifications on the melt-curve analysis. Here, the melt curve was prepared by ramping up the melting temperature from 60 °C to 95 °C at a ramp rate of 0.15 at each step on a continuous mode following a pre-melt step at 95 °C for 15s on the 1st step. All reactions were performed on a QuantStudio 5 Real-Time PCR System (ThermoFisher Scientific, Lenexa KS, USA). The melt-curve analysis was carried out using the QuantStudio Design and Analysis software version 1.4.3 (ThermoFisher Scientific, Lenexa KS, USA). *C. jejuni* ATCC 33560 and *C. coli* ATCC 33559 were used as positive controls while the reaction mixture without template DNA was used as a negative control.

### 4.6. Antibiotic Susceptibility Testing of Isolates

The antibiotic susceptibility profiles of the isolates were determined using the Kirby–Bauer disc diffusion method, and the results were interpreted using the Clinical and Laboratory Standards Institute guidelines [39]. Antibiotics tested included clindamycin (10 µg), erythromycin (15 µg), ampicillin (10 µg), ciprofloxacin (5 µg), nalidixic acid (30 µg), gentamicin (10 µg), ceftriaxone (30 µg) and tetracycline (30 µg) as recommended by the WHO AGISAR guidelines. A *Campylobacter* spp. suspension equivalent to a 0.5 McFarland standard (measured using a McFarland densitometer [DEN 1B, Bioscan]) as recommended by the CLSI (Clinical & Laboratory Standards Institute) guidelines was spread on Mueller–Hinton Agar (Oxoid, Hampshire, UK) supplemented with 5% defibrinated sheep blood. Antibiotic discs were aseptically placed onto the inoculated agar plates and incubated at 37 °C for 48 h under microaerophilic conditions. Antibiotic susceptibility was calculated by the zone of clearance observed around each antibiotic disc in millimeters. ATCC 33560 (*C. jejuni*) and ATCC 33559 (*C. coli*) were used as controls.

### 4.7. Molecular Detection and DNA Sequence Analysis of Genetic Determinants of Resistance

Extracted DNA was used to detect the presence of genes and chromosomal mutations conferring resistance to antibiotics using real-time PCR. The primer sequences and PCR conditions are shown in Table 3.

Primers were purchased from Inqaba Biotechnical Industries (Pty) Ltd., Pretoria, South Africa. The presence of erythromycin resistance was determined by detecting point mutations at positions 2074 and 2075 in domain V of the *23S rRNA.* Thr-86-Ile mutations found in the quinolone resistance determining region (QRDR) of the *gyrA* gene in *Campylobacter* were used to predict for fluoroquinolone resistance. Tetracycline and multidrug efflux pump-mediated resistance were detected using the *tet(O*) and *cmeB* genes, respectively.

Sequencing of selected amplified PCR products was used to confirm the acquired resistance (*tet(O*) and *cmeB*) and chromosomal mutations (*23S rRNA* and *gyrA*) in the *Campylobacter* isolates. Sequence reaction, purification, and analysis were done on an ABI 3130XL Genetic analyzer using the Sanger method of DNA sequencing carried out at Inqaba Biotechnical Industries (Pty) Ltd., Pretoria, South Africa. The sequences were analysed using the Basic Local Alignment Search Tool^®^ 2.0 software, available from the National Center for Biotechnology Information (http://www.ncbi.nhlm.nih.gov/blast/BLAST.cgi).

### 4.8. Detection of Virulence Genes

Real-time PCR was used to test for the presence of virulence genes involved in adhesion (*cadF, pldA*), invasion (*ciaB*), toxin production (*cdtA, cdtB*, *cdtC*) and thermo-tolerance (*dnaJ*) in the isolates. The reaction was carried out in a total volume of 20 µL made up of 10 µL of a New England Biolabs^®^ 2× Luna^®^ Universal qPCR master mix (Inqaba Biotechnical Industries (Pty) Ltd., Pretoria, South Africa), 0.5 µL of each primer (final concentration 0.5 µM), 5 µL of template DNA and 1 µL of nuclease-free water. The PCR conditions were as previously optimised by Chukwu et al. [38] with slight modifications on the melt-curve analysis. Here, the melt curve was prepared by ramping up the melting temperature from 60 °C to 95 °C at a ramp rate of 0.15 at each step on a continuous mode following a pre-melt step at 95 °C for 15 s on the 1st step. All reactions were performed on a QuantStudio 5 Real-Time PCR System (ThermoFisher Scientific, Lenexa KS, USA). The melt-curve analysis was carried out using the QuantStudio Design and Analysis software version 1.4.3 (ThermoFisher Scientific, Lenexa KS, USA). *C. jejuni* ATCC 33560 and *C. coli* ATCC 33559 were used as positive controls while the reaction mixture without template DNA was used as a negative control. All samples were run in triplicate. The conditions for the confirmation of virulence genes in the isolates have been shown in Table 4.

### 4.9. Genetic Relationship by Pulsed-Field Gel Electrophoresis (PFGE)

A sub-sample of isolates representative of the source and antibiograms was chosen to determine the clonality of the isolates using PFGE. The PulseNet protocol by Ribot et al. [42] was used with slight modifications. In brief, a cell suspension of an optical density of 1.3 at 610 nm was embedded in 1% low melting agarose (Bio-Rad, Hercules CA, USA). The DNA was digested with 2 µL of *Sma1* (Bio-Labs, New England) per sample and incubated for 2 h at 25 °C. Macro-restriction fragments were separated by electrophoresis using CHEF Mapper^®^ (Bio-Rad, Hercules, CA, USA) in 1% SeaKem Gold Agarose (VWR Life Science, UK) gel at 6 V/cm at 14 °C for 19 h. *Salmonella* serotype Braenderup H9812 strain digested by *Sma1* was used as a run control. Images were analysed with the Bionumerics software (Applied Maths, Austin TX, USA). Optimization and band tolerance were set at 1% (version 7.6, Applied Maths, Austin TX, USA) and 80% similarity cut-off was used to define clusters or pulse types.

### 4.10. Data Analysis and Interpretation

The data were analysed using GraphPad Prism statistical software package (GraphPad Prism v5; Software Inc., San Diego, CA, USA). Descriptive statistics were used to describe the frequency of *Campylobacter* spp. that was isolated from different samples and sources.

## 5. Conclusions

To the best of our knowledge, this is the first study of *Campylobacter* spp. from farm-to-fork in intensive poultry farming systems in the KwaZulu-Natal Province characterising resistance, virulence factors, and their genetic relatedness. The numerous permutations of resistance genes, virulence factors, and clonal relatedness in the isolates irrespective of various sources point to complex molecular epidemiology and aetiology. It should, however, be noted that the presence of a gene does not necessarily imply the expression of that gene. Therefore, although different resistance and virulence genes were identified in this study, it would be important to confirm expression of these genes in isolates from intensive poultry farming to better understand the potential impact these could have on food safety and public health. Proper preventative measures are needed to prevent contamination of poultry at both the production and retail level.

## Figures and Tables

**Table 1:** Antibiotic resistance profiles.

Resistance Profiles	No. *C. jejuni* Isolates (*n* = 45) (%)	No. *C. coli* Isolates (*n* = 60) (%)
AMP-NAL-ERY-GEN-CRO-CLI	8 (19.04)	1 (1.67)
AMP-NAL-CIP-ERY-CRO-CLI	11 (26.19)	8 (13.3)
AMP-NAL-CIP-CRO-CLI	3 (7.14)	0
AMP-NAL-ERY-GEN-CLI	0	1 (1.67)
AMP-NAL-ERY-CRO-CLI	2 (4.76)	0
AMP-NAL-ERY-TET-CRO-CLI	3 (6.7)	1
AMP-CIP-ERY-CRO	1 (2.3)	0
AMP-NAL-ERY-CRO	1 (2.3)	0
NAL-CIP-ERY-CRO	2 (4.76)	0
AMP-NAL-ERY-CLI	0	3 (5.00)
AMP-CIP-ERY-CLI	0	1 (1.67)
AMP-ERY-GENT-CLI	1 (2.3)	1 (1.67)
NAL-ERY-GENT-CLI	1 (2.3)	0
AMP-NAL-ERY-GENT	1 (2.3)	0
AMP-ERY-CRO-CLI	0	1 (1.67)
ERY-CRO-CLI	0	1 (1.67)
AMP-NAL-CRO	1 (2.3)	3 (5.00)
AMP-NAL-ERY	1 (2.3)	0
ERY-GENT- CLI	0	1 (1.67)
AMP-ERY-CLI	0	2 (3.33)
NAL-ERY-CLI	0	1 (1.67)
CIP-ERY-GENT	0	1 (1.67)
ERY-CLI	7 (16.67)	14 (23.33)
AMP-CLI	0	2 (3.33)
AMP-GENT	0	2 (3.33)
AMP-ERY	0	3 (5.00)
AMP- CRO	1 (2.3)	0
ERY	3 (7.14)	4 (26.67)
CRO	0	3
CLI	0	4 (26.67)
AMP	0	2 (3.33)

**Table 2:** Primers used for the confirmation of *Campylobacter* genus and species.

Target Gene	Primer Sequence (5′–3′)	Product Size (bp)	Annealing Temperature (°C)	References
*16S rRNA*	F-GGATGACACTTTTCGGAGC R-CATTGTAGCACGTGTGTC	816	58	[36]
*Asp*	F-GGTATGATTTCTACAAAGCGAG R-ATAAAAGACTATCGTCGCGTG	500	53	[37]
*hipO*	F-GAAGAGGGTTTGGGTGGT R-AGCTAGCTTCGCATAATAACTTG	735	53	[37]

**Table 3:** Primers used for the detection of antibiotic resistance genes and point mutations.

Target Gene	Primer Sequence (5′–3′)	Product Size (bp)	Annealing Temperature (°C)	References
*cmeB*	F5′GACGTAATGAAGGAGAGCCA R5′CTGATCCACTCCAGCTATG	1166	50	[40]
*tet(O)*	F-GGCGTTTTGTTTATGTGCG R-ATGGACAACCCGACAGAAGC	559	49	[5]
*23S rRNA at position 2074*	F5′TTAGCTAATGTTGCCCGTACCG R5′AGTAAAGGTCCACGGGGTCTCG	485	59	[38]
*23S rRNA at position 2075*	F5′TTAGCTAATGTTGCCCGTACCG R5′TAGTAAAGGTCCACGGGGTCGC	485	59	[38]
*gyrA*	F-GAAGAATTTTATATGCTATG R-TCAGTATAAC GCATCGCAGC	235	53	[30]
*gyrA*	F-ACGCAAGAGAGATGGTT R-TCAGTATAACGCATCGCAGC	270	45	[30]

**Table 4:** Primers used for the detection of virulence genes.

Target Gene	Primer Sequence (5′–3′)	Product Size (bp)	Annealing Temperature (°C)	References
*cadF*	F-TTGAAGGTAATTTAGATATG R-CTAATACCTAAAGTTGAAAC	400	43	[41]
*ciaB*	F-TGCGAGATTTTTCGAGAATG R-TGCCCGCCTTAGAACTTACA	527	54	[41]
*dnaJ*	F-ATTGATTTTGCTGCGGGTAG R-ATCCGCAAAAGCTTCAAAAA	177	50	[41]
*pldA*	F-AAGAGTGAGGCGAAATTCCA R-GCAAGATGGCAGGATTATCA	385	46	[41]
*cdtA*	F-CCTTGTGATGCAAGCAATC R-ACACTCCATTTGCTTTCTG	370	49	[21]
*cdtB*	F-GTTAAAATCCCCTGCTATCAACCA R-GTTGGCACTTGGAATTTGCAAGGC	495	51	
*cdtC*	F-CGATGAGTTAAAACAAAAAGATA R-TTGGCATTATAGAAAATACAGTT	182	48	

## Supplementary Materials

The following is available online at https://www.mdpi.com/2079-6382/9/2/42/s1, Table S1: Antibiogram of individual *Campylobacter* isolates.

## References

[1] Umaraw P., Prajapati A., Verma A.K., Pathak V., Singh V.P., Umaraw P., Prajapati A., Verma A.K., Pathak V. (2017). Control of campylobacter in poultry industry from farm to poultry processing unit: A review. Crit. Rev. Food Sci. Nutr..

[2] Alanis A.J. (2005). Resistance to antibiotics: Are we in the post-antibiotic era?. Arch. Med. Res..

[3] Butzler J.-P. (2004). Campylobacter, from obscurity to celebrity. Clin. Microbiol. Infect..

[4] World Health Organization (WHO) Campylobacter. https://www.who.int/news-room/fact-sheets/detail/campylobacter.

[5] Reddy S., Zishiri O.T. (2017). Detection and prevalence of antimicrobial resistance genes in Campylobacter spp. isolated from chickens and humans. Onderstepoort J. Veter. Res..

[6] Van Boeckel T.P., Brower C., Gilbert M., Grenfell B.T., Levin S.A., Robinson T.P., Teillant A., Laxminarayan R. (2015). Global trends in antimicrobial use in food animals. Proc. Natl. Acad. Sci. USA.

[7] Sibanda N., McKenna A., Richmond A., Ricke S.C., Callaway T., Stratakos A.C., Gundogdu O., Corcionivoschi N. (2018). A Review of the Effect of Management Practices on Campylobacter Prevalence in Poultry Farms. Front. Microbiol..

[8] Hermans D., Van Deun K., Martel A., Van Immerseel F., Messens W., Heyndrickx M., Haesebrouck F., Pasmans F. (2011). Colonization factors of Campylobacter jejuni in the chicken gut. Veter. Res..

[9] Wegener H.C. (2003). Antibiotics in animal feed and their role in resistance development. Curr. Opin. Microbiol..

[10] Maćkiw E., Korsak D., Rzewuska K., Tomczuk K., Rożynek E. (2012). Antibiotic resistance in Campylobacter jejuni and Campylobacter coli isolated from food in Poland. Food Control.

[11] Bolton D.J. (2015). Campylobacter virulence and survival factors. Food Microbiol..

[12] Semenya M. (2018). Poultry Industry: South African Poultry Association Briefing. DAFF 2018/19 Annual Performance Plan.

[13] Viljoen W. (2017). The South African Poultry Sector—Trade, Consumption, Production and Inputs.

[14] WHO (2017). Integrated Surveillance of Antimicrobial Resistance in Foodborne Bacteria: Application of a One Health Approach: Guidance from the WHO Advisory Group on Integrated Surveillance of Antimicrobial Resistance (AGISAR).

[15] Amoako D.G., Somboro A.M., Abia A.L.K., Allam M., Ismail A., Bester L., Essack S.Y. (2019). Genomic analysis of methicillin-resistant Staphylococcus aureus isolated from poultry and occupational farm workers in Umgungundlovu District, South Africa. Sci. Total Environ..

[16] Molechan C., Amoako D.G., Abia A.L.K., Somboro A.M., Bester L.A., Essack S.Y. (2019). Molecular epidemiology of antibiotic-resistant Enterococcus spp. from the farm-to-fork continuum in intensive poultry production in KwaZulu-Natal, South Africa. Sci. Total Environ..

[17] Newell D.G., Fearnley C. (2003). Sources of *Campylobacter* Colonization in Broiler Chickens. Appl. Environ. Microbiol..

[18] Willis W.L., Murray C. (1996). Campylobacter jejuni Seasonal Recovery Observations of Retail Market Broilers 1. Poult. Sci..

[19] Powell L., Lawes J., Clifton-Hadley F., Rodgers J., Harris K., Evans S., Vidal A. (2019). The prevalence of Campylobacter spp. in broiler flocks and on broiler carcases, and the risks associated with highly contaminated carcases. Epidemiol. Infect..

[20] Wieczorek K., Osek J. (2008). Identification of Virulence Genes in Campylobacter Jejuni and C. Coli Isolates by PCR. Bull. Vet. Inst. Pulawy.

[21] Rizal A., Kumar A., Vidyarthi A.S. (2010). Prevalence of Pathogenic Genes in Campylobacter jejuni Isolated from Poultry and Human. Internet J. Food Saf..

[22] Carvalho A.F.D., Martins D., Azevedo S.S., Piatti R.M., Genovez M.E., Scarcelli E. (2013). Detection of CDT toxin genes in Campylobacter spp. strains isolated from broiler carcasses and vegetables in São Paulo, Brazil. Braz. J. Microbiol..

[23] Lapierre L., Gatica M.A., Riquelme V., Vergara C., Yañez J.M., San Martín B., Sáenz L., Vidal M., Martínez M.C., Araya P. (2016). Characterization of Antimicrobial Susceptibility and Its Association with Virulence Genes Related to Adherence, Invasion, and Cytotoxicity in Campylobacter jejuni and Campylobacter coli Isolates from Animals, Meat, and Humans. Microb. Drug Resist..

[24] Wieczorek K. (2010). Antimicrobial resistance and virulence markers of campylobacter jejuni and campylobacter coli isolated from retail poultry meat in Poland. Bull. Vet. Inst. Pulawy.

[25] Koolman L., Whyte P., Burgess C., Bolton D. (2016). International Journal of Food Microbiology Virulence gene expression, adhesion and invasion of Campylobacter jejuni exposed to oxidative stress (H_2_O_2_). Int. J. Food Microbiol..

[26] Wareing D.R.A., Kramer J.M., Frost J.A., Bolton F.J. (2000). Campylobacter Contamination of Raw Meat and Poultry at Retail Sale: Identification of Multiple Types and Comparison with Isolates from Human Infection. J. Food Prot..

[27] Melo R.T., Nalevaiko P.C., Mendonça E.P., Borges L.W., Fonseca B.B., Beletti M.E., Rossi D.A. (2013). Campylobacter jejuni strains isolated from chicken meat harbour several virulence factors and represent a potential risk to humans. Food Control.

[28] Di Sario G. (2017). Investigation of the Effects of Campylobacter iejuni Virulence Factors in Human Cells: Different Pathways Involved. Ph.D. Thesis.

[29] Baserisalehi M., Bahador N. (2011). Anaerobe Chemotactic behavior of Campylobacter spp. in function of different temperatures. Anaerobe.

[30] Shobo C.O., Bester L.A., Baijnath S., Somboro A.M., Peer A.K., Essack S.Y. (2016). Antibiotic resistance profiles of Campylobacter species in the South Africa private health care sector. J. Infect. Dev. Ctries..

[31] Denis M., Rose V., Balaine L., Salvat G. (2007). Diversity of Pulsed-Field Gel Electrophoresis Profiles of Campylobacter jejuni and Campylobacter coli from Broiler Chickens in France. Poult. Sci..

[32] Bester L.A., Essack S.Y., Essack S. (2008). Prevalence of antibiotic resistance in Campylobacter isolates from commercial poultry suppliers in KwaZulu-Natal, South Africa. J. Antimicrob. Chemother..

[33] Gibreel A., Tracz D.M., Nonaka L., Ngo T.M., Connell S.R., Taylor D.E. (2004). Incidence of Antibiotic Resistance in Campylobacter jejuni Isolated in Alberta, Canada, from 1999 to 2002, with Special Reference to tet(O)-Mediated Tetracycline Resistance. Antimicrob. Agents Chemother..

[34] Gardner S.P., Kendall K.J., Taveirne M.E., Olson J.W. (2017). Complete Genome Sequence of Campylobacter jejuni subsp. jejuni ATCC 35925. Genome Announc..

[35] Linton D., Lawson A.J., Owen R.J., Stanley J. (1997). PCR detection, identification to species level, and fingerprinting of Campylobacter jejuni and Campylobacter coli direct from diarrheic samples. J. Clin. Microbiol..

[36] Amri A.A., Senok A.C., Ismaeel A.Y., Al-mahmeed A.E., Botta G.A. (2019). Multiplex PCR for direct identification of Campylobacter spp. in human and chicken stools. J. Med. Microbiol..

[37] Chukwu M.O., Abia A.L.K., Ubomba-Jaswa E., Obi L., Dewar J.B. (2019). Characterization and Phylogenetic Analysis of Campylobacter Species Isolated from Paediatric Stool and Water Samples in the Northwest Province, South Africa. Int. J. Environ. Res. Public Health.

[38] Clinical and Laboratory Standards Institute (2017). Performance Standards for Antimicrobial Susceptibility Testing: 27th Edition Informational Supplement M100-S27.

[39] Huq M., Gonis G., Istivan T. (2014). Development and Evaluation of a Multiplex PCR for the Detection of Campylobacter concisus and Other Campylobacter spp. from Gastroenteritis Cases. Open J. Med. Microbiol..

[40] Epps S.V.R., Harvey R.B., Hume M.E., Phillips T.D., Anderson R.C., Nisbet D.J. (2013). Foodborne Campylobacter: Infections, Metabolism, Pathogenesis, and Reservoirs. Int. J. Environ. Res. Public Health.

[41] Chansiripornchai N., Sasipreeyajan J. (2009). PCR detection of four virulence-associated genes of Campylobacter jejuni isolates from Thai broilers and their abilities of adhesion to and invasion of INT-407 cells. J. Veter. Med. Sci..

[42] Ribot E.M., Fitzgerald C., Kubota K., Swaminathan B., Barrett T.J. (2001). Rapid Pulsed-Field Gel Electrophoresis Protocol for Subtyping of Campylobacter jejuni. J. Clin. Microbiol..

